# Longitudinal assessment of real-world patient adherence: a 12-month electronic patient-reported outcomes follow-up of women with early breast cancer undergoing treatment

**DOI:** 10.1007/s00520-024-08547-7

**Published:** 2024-05-14

**Authors:** Pimrapat Gebert, Anna Maria Hage, Jens-Uwe Blohmer, Robert Roehle, Maria Margarete Karsten

**Affiliations:** 1 Berlin Institute of Health at Charité –Universitätsmedizin Berlin, Berlin, Germany; 2 Institute of Biometry and Clinical Epidemiology, Charité – Universitätsmedizin Berlin, Berlin, Germany; 3 Department of Gynecology With Breast Center, Charité – Universitätsmedizin Berlin, Berlin, Germany

**Keywords:** Oncology, Web-based questionnaire, Return rate, Compliance, Early Breast cancer

## Abstract

**Background:**

Electronic patient-reported outcomes (ePROs) assess patients’ health status and quality of life, improving patient care and treatment effects, yet little is known about their use and adherence in routine patient care.

**Aims:**

We evaluated the adherence of invasive breast cancer and ductal carcinoma in situ (DCIS) patients to ePROs follow-up and whether specific patient characteristics are related to longitudinal non-adherence.

**Methods:**

Since November 2016, the Breast Center at Charité – Universitätsmedizin Berlin has implemented an ongoing prospective *PRO routine* program, requiring patients to complete ePROs assessments and consent to email-based follow-up in the first 12 months after therapy starts. Frequencies and summary statistics are presented. Multiple logistic regression models were performed to determine an association between patient characteristics and non-adherence.

**Results:**

Out of 578 patients, 239 patients (41.3%, 95%CI: 37.3–45.5%) completed baseline assessment and all five ePROs follow-up during the first 12 months after therapy. On average, above 70% of those patients responded to the ePROs follow-up assessment. Adherence to the ePROs follow-up was higher during the COVID-19 pandemic than in the time periods before (47.4% (111/234) vs. 33.6% (71/211)). Factors associated with longitudinal non-adherence were younger age, a higher number of comorbidities, no chemotherapy, and a low physical functioning score in the EORTC QLQ-C30 at baseline.

**Conclusions:**

The study reveals moderate adherence to 12-month ePROs follow-up assessments in invasive early breast cancer and DCIS patients, with response rates ranging from 60 to 80%. Emphasizing the benefits for young patients and those with high disease burdens might further increase adherence.

**Supplementary Information:**

The online version contains supplementary material available at 10.1007/s00520-024-08547-7.

## Introduction

The utilization of digital health technologies on smartphones, tablets, and computers for the purposes of symptom monitoring and assessing quality of life throughout the follow-up period has increased rapidly [1]. In oncological care, electronic patient-reported outcomes (ePROs) have been extensively studied to enable patients to self-report symptoms and quality of life, as well as to monitor patients during oncological treatment [2–6]. It is important to note that the ePROs are valuable resources when reviewed and used by healthcare professionals, highlighting the crucial role of professional interpretation and utilization. Patient-reported outcomes (PROs) refer to any report of a patient’s health status that comes directly from the patient without interpretation by a clinician or anyone else [7]. The European Organization for Research and Treatment of Cancer’s Quality of Life Questionnaire (EORTC QLQ-C30) [8] is one of the widely used instruments assessing PROs in oncological research. Numerous studies have demonstrated that continuously monitoring ePROs not only enhances the accuracy of symptom assessment and functional status of patients [2, 9, 10], but also leads to improved patient-clinician communication [11]. However, despite these advantages, the widespread integration of ePROs into routine clinical practice remains limited, and the precise factors influencing their adoption and efficacy in clinical settings are not fully understood.

Adherence rates in clinical cancer trials that measured ePROs ranged from 65% to more than 90% [12, 13]. For example, 83% of breast cancer patients responded when assessing ePROs during adjuvant radiotherapy [14], and more than 90% of advanced and metastatic cancer patients completed ePROs [15]. However, in non-clinical trial settings, decreasing adherence in follow-up ePROs assessments was reported, with lower rates around 40–60% [16, 17], depending on the number of follow-up assessments and the presence of reminder systems [18].

While numerous studies have demonstrated the advantages of using ePROs in clinical care [17, 19], their integration into routine clinical settings has progressed slower than anticipated. A systematic review examining factors influencing adherence to ePROs in patients with chronic diseases revealed that symptom severity, comorbidity, marital status, electronic health literacy and satisfaction with using ePROs may be associated with higher adherence, although the evidence was inconclusive for any identified factors [20]. Therefore, there remains limited understanding of the factors influencing patient adherence over a long follow-up period. This study aims to evaluate these factors by employing process and outcome measures to analyze adherence to ePROs over time among breast cancer (including ductal carcinoma in situ (DCIS)) patients in the *PRO routine* program at the Breast Center of the Charité – Universitätsmedizin Berlin [18]. Identifying the factors associated with non-adherence can facilitate the identification of patients who may require additional education about the benefits of ePROs. Additionally, we aim to evaluate the impact of the COVID-19 pandemic on patient adherence to ePROs follow-up.

In this study, several key outcome measures are used to assess patient adherence and response rates to ePROs assessments:

- *12-month adherence:* Patients who completed all five follow-up ePRO assessments during the first 12 months following their treatment and the baseline assessment.
- *Non-adherence:* Patients who missed at least one of the follow-up ePRO assessments during the first 12 months following their treatment or the baseline assessment.
- *Response rate:* The percentage of patients who returned the questionnaires for each time point of the follow-up ePRO assessment.

## Methods

Since November 2016, the Breast Center at Charité – Universitätsmedizin Berlin has implemented an ongoing prospective *PRO routine* program, using the International consortium for Health Outcome Measurement (ICHOM) breast cancer data set [18]. This program has received ethics approval from the Charité Ethics Commission (EA 4/127/16). The *PRO routine* program is offered to every new patient visiting the breast center. Patients complete baseline assessments on a tablet computer in the clinic before their appointment and have the option to consent to further participation in an e-mail-based follow-up. The ePRO follow-up is initiated at the time of their initial surgery or start of chemotherapy, whichever comes first, by the study team. During the first 12 months after therapy, patients are followed up five times. However, patients who undergo breast conserving surgery have an additional assessment at 2 weeks (Additional Table S1). Patients receive an email with a link to the questionnaires and up to three automated reminders if the assessment is not completed. After 12 months, patients are followed up on a half-yearly basis, and after 5 years, on a yearly basis. However, in this study, we only assessed adherence during the first year of follow-up.

### Study sample

**Fig. 1:**
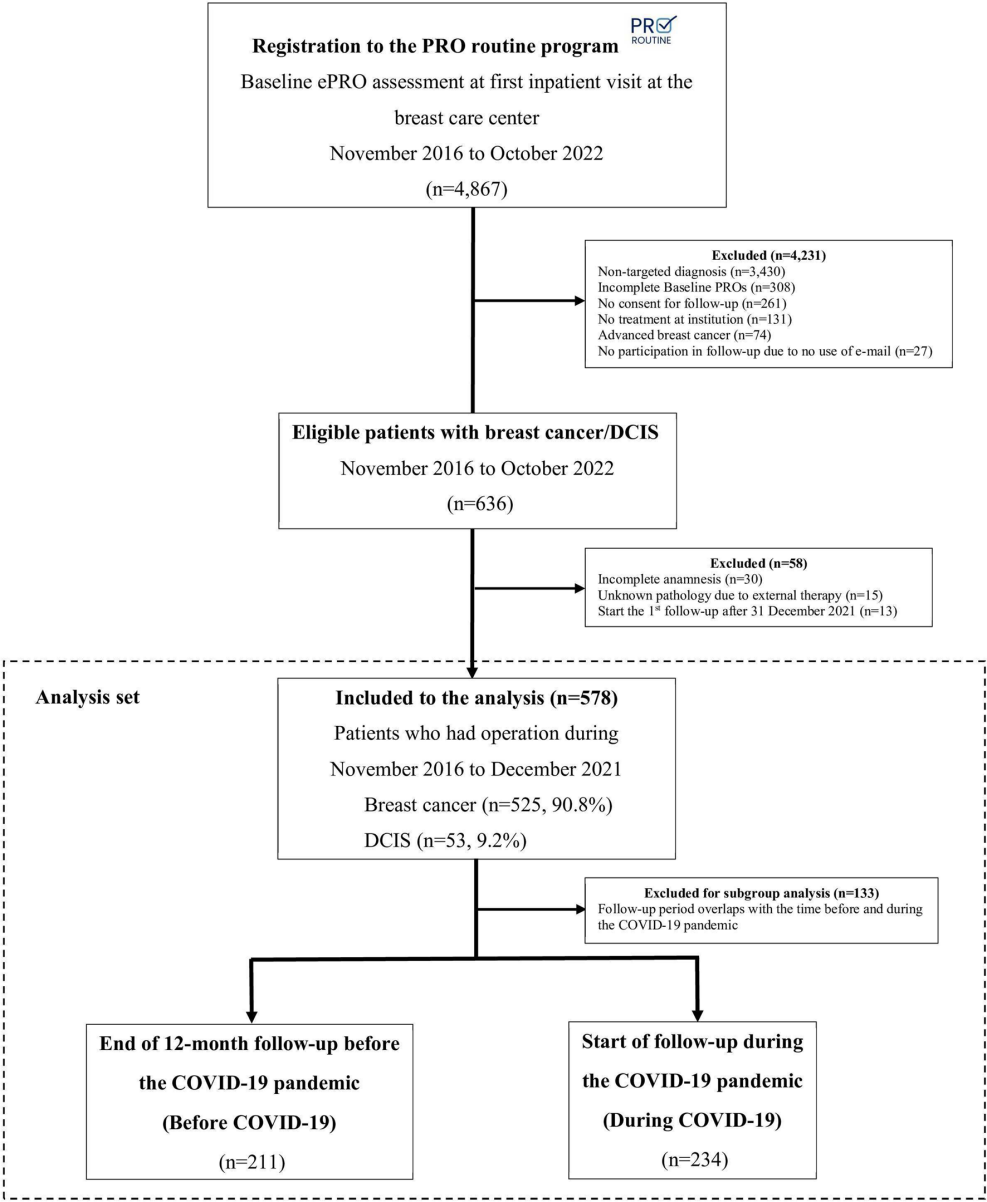
Study flowchart with the inclusion process. DCIS = Ductal carcinoma in situ; PROs = Patient-reported outcomes

Between November 2016 and October 2022, a total of 4,867 patients from the breast cancer clinic were registered in the *PRO Routine* system. However, 4,231 (86.9%) patients were excluded because they did not meet inclusion criteria for this analysis. Among the excluded patients, 81% did not receive a targeted diagnosis such as fibroadenoma, cysts, mastopathy, or abscesses. Further exclusion criteria are detailed in Fig. 1. Patients who registered for our *PRO Routine* assessment, were diagnosed with breast cancer or DCIS, and underwent surgery at the Charité Breast Center between November 2016 and December 31st, 2021 were included in this study. The follow-up period for analyzing the collected ePROs ended on December 31st, 2022.

In addition to the more general questions regarding adherence, we sought to assess the impact of the COVID-19 pandemic on adherence to ePRO follow-up. Consequently, we divided patients into two cohorts based on the onset of the first lock-down in Germany (March 22nd, 2020): one comprising patients before the pandemic (*n* = 211), and the other during the pandemic, spanning from March 22nd, 2020, to December 31st, 2020 (*n* = 234). We aimed to compare adherence levels between these two periods. Patients with overlapped follow-up periods before and during the pandemic were excluded from this subgroup analysis (*n* = 133).

Given the exploratory nature of this study, we did not provide a formal power statement.

### Data collection

Demographic data and medical history were collected electronically with self-reported baseline patient questionnaires at the time of registration to the *PRO Routine* program, e.g., age, weight, height, comorbidities, history of cancer, family history of breast or ovarian cancer, previous breast surgery, family status, and education. Tumor stage was classified using the 8th edition of the TNM classification (TNM8) [21]. Education was classified into three groups: low (secondary school graduation or less), high (high school graduation or more) or medium (anything in between). We assessed PROs using the EORTC QLQ-C30 (version 3.0) [8]. This instrument consists of nine multi-item scales: five functional scales (physical, role, cognitive, emotional, and social functioning); three symptoms scales (fatigue, pain, and nausea/vomiting); six single-item scales (dyspnoea, insomnia, appetite loss, constipation, diarrhea, and financial difficulties); and a global health status/QoL scale. The total score is calculated by averaging items within scales and transforming them to the range from 0 to 100. High values of a functional scale represent a good functioning, whereas high values of a symptom scale or item represent a high symptom burden.

### Statistical analysis

Frequencies and summary statistics were presented separately by adherence and non-adherence groups. Multiple logistic regression models were performed to determine the association between patient characteristics and non-adherence. Factors included age at the registration [in years], education level [low, medium, high], marital status [single, married/partnership, divorced/separated/widowed], number of comorbidities [< 2 and ≥ 2 comorbidities], self-assessment of difficulty filling out the baseline questionnaires (scores range 1–6 = very difficult), preference for paper-based PRO assessment at baseline [yes, no], first breast cancer disease [yes, no], type of surgery [breast conserving surgery, mastectomy with reconstruction, mastectomy without reconstruction], chemotherapy [yes, no], and continuous variables of the EORTC QLQ C-30 at baseline. Adjusted odds ratio (aOR) with 95% confidence intervals (CIs) was reported. The Akaike information criterion (AIC) was considered when comparing the model selection, and the Hosmer–Lemeshow test was performed for goodness of fit for the final model. Missing data is assumed to be completely at random; hence, no imputation method is applied given the low rate of missing data. Furthermore, binary variables indicating better or worsening scores for each subscales of the EORTC QLQ C-30 at baseline were generated using cut-points derived from reference values in Germany [22]. A generalized linear model employing a binomial family and a log link function was conducted to estimate relative risk (RR) and 95%CI to explore the association between binary variables of the EORTC QLQ C-30 and adherence. Statistical testing was done within an exploratory framework at a two-sided significance level of *α* = 0.05. All the statistical tests were performed using Stata IC15 (StataCorp, 2017, College Station, TX, USA).

## Results

A total of 578 patients with invasive breast cancer (525, 90.8%) and DCIS (53, 9.2%) participated in the analysis of adherence to e-mail-based ePRO follow-up during 12 months post-therapy as part of the *PRO routine* program at the Breast Center of Charité – Universitätsmedizin Berlin (Fig. 1). The program includes an ePRO assessment at baseline and five subsequent follow-ups during the initial 12 months post-therapy (Supplementary Information Table S1).

Table 1 provides an overview of the patients’ characteristics. The mean age at registration was 54 years (range: 23–88 years). Most of the participants had attained a high level of education and were either married or in partnerships. Among the cohort, 68% (393/578) underwent breast-conserving surgery, and 45.0% (260/578) received chemotherapy. The median follow-up time was 25 months (interquartile range (IQR): 13–40 months).

**Table 1:** Socio-demographic and health characteristics of the patient’s study cohort

Patient characteristics	Total (*n* = 578)	Adherence (*n* = 239)	Non-adherence (*n* = 339)	*p*-value
Age at registration, mean (SD) [min, max]	54 (12) [23, 88]	53 (12) [24, 79]	54 (12) [23, 88]	0.37
< 40	63 (10.9%)	27 (11.3%)	36 (10.6%)	
40–49	150 (26.0%)	62 (25.9%)	88 (26.0%)	
50–59	179 (31.0%)	79 (33.1%)	100 (29.5%)	
≥ 60	186 (32.2%)	71 (29.7%)	115 (33.9%)	
Educational level	*n* = 577	*n* = 239	*n* = 338	0.040
Low	33 ( 5.7%)	7 ( 2.9%)	26 ( 7.7%)	
Medium	140 (24.3%)	56 (23.4%)	84 (24.9%)	
High	404 (70.0%)	176 (73.6%)	228 (67.5%)	
BMI (kg/m^2^), mean (SD)	24.9 (5.0)	24.6 (4.7)	25.2 (5.2)	0.22
Marital status	*n* = 543	*n* = 236	*n* = 307	0.043
Single	67 (12.3%)	32 (13.6%)	35 (11.4%)	
Married/partnership	398 (73.3%)	181 (76.7%)	217 (70.7%)	
Divorced/separated	55 (10.1%)	18 ( 7.6%)	37 (12.1%)	
Widowed	23 ( 4.2%)	5 ( 2.1%)	18 ( 5.9%)	
Menopause	224 (38.8%)	93 (38.9%)	131 (38.6%)	0.95
Alcohol consumption				0.004
None	150 (26.0%)	45 (18.8%)	105 (31.0%)	
Occasionally	332 (57.4%)	153 (64.0%)	179 (52.8%)	
Weekly	77 (13.3%)	30 (12.6%)	47 (13.9%)	
Daily	19 ( 3.3%)	11 ( 4.6%)	8 ( 2.4%)	
Smoking status				0.66
No	414 (71.6%)	176 (73.6%)	238 (70.2%)	
Current smoker	90 (15.6%)	35 (14.6%)	55 (16.2%)	
Ex-smoker	74 (12.8%)	28 (11.7%)	46 (13.6%)	
Comorbidities				0.003
None	287 (49.7%)	130 (54.4%)	157 (46.3%)	
One comorbidity	161 (27.9%)	72 (30.1%)	89 (26.3%)	
Two or more comorbidities	130 (22.5%)	37 (15.5%)	93 (27.4%)	
Self-assessment of difficulties completing the baseline PRO questionnaires (1 = not difficult – 6 = very difficult), mean (SD)	2.0 (1.1)	1.9 (1.0)	2.1 (1.2)	0.037
Preference for paper-based PRO questionnaires at baseline	99 (17.1%)	35 (14.6%)	64 (18.9%)	0.18
Previous breast cancer	82 (14.2%)	26 (10.9%)	56 (16.5%)	0.056
Diagnosis				0.021
Breast cancer	525 (90.8%)	225 (94.1%)	300 (88.5%)	
DCIS	53 ( 9.2%)	14 ( 5.9%)	39 (11.5%)	
Type of surgery				0.22
Breast-conserving surgery	393 (68.0%)	161 (67.4%)	232 (68.4%)	
Mastectomy with reconstruction	135 (23.4%)	62 (25.9%)	73 (21.5%)	
Mastectomy without reconstruction	50 ( 8.7%)	16 ( 6.7%)	34 (10.0%)	
Any chemotherapy	260 (45.0%)	128 (53.6%)	132 (38.9%)	< 0.001
Any targeted therapy	80 (13.8%)	35 (14.6%)	45 (13.3%)	0.64
Any radiation therapy	406 (70.2%)	171 (71.5%)	235 (69.3%)	0.56
Any endocrine therapy	369 (63.8%)	158 (66.1%)	211 (62.2%)	0.34
Tumor stage				0.19
0	127 (22.9%)	49 (21.3%)	78 (24.0%)	
1	209 (37.7%)	78 (33.9%)	131 (40.3%)	
2	177 (31.9%)	83 (36.1%)	94 (28.9%)	
3	42 ( 7.6%)	20 ( 8.7%)	22 ( 6.8%)	
Histological Subtype				0.086
Ductal carcinoma in situ	55 ( 9.5%)	16 ( 6.7%)	39 (11.5%)	
Invasive ductal carcinoma	424 (73.4%)	185 (77.4%)	239 (70.5%)	
Invasive lobular carcinoma	63 (10.9%)	23 ( 9.6%)	40 (11.8%)	
Other	32 ( 5.5%)	15 ( 6.3%)	17 ( 5.0%)	
Unknown	4 ( 0.7%)	0 ( 0.0%)	4 ( 1.2%)	
Tumor grading				0.83
Grade 1 (low)	85 (16.2%)	40 (17.8%)	45 (15.1%)	
Grade 2 (intermediate)	297 (56.7%)	124 (55.1%)	173 (57.9%)	
Grade 3 (high)	125 (23.9%)	53 (23.6%)	72 (24.1%)	
Unknown	17 ( 3.2%)	8 ( 3.6%)	9 ( 3.0%)	
DCIS-Grading				0.045
Grade 1 (low)	10 (18.9%)	6 (42.9%)	4 (10.3%)	
Grade 2 (intemediate)	19 (35.8%)	3 (21.4%)	16 (41.0%)	
Grade 3 (high)	21 (39.6%)	5 (35.7%)	16 (41.0%)	
Unknown	3 ( 5.7%)	0 ( 0.0%)	3 ( 7.7%)	
Median ePRO follow-up time (months) (IQR)	25.0 (13.0–40.0)	26.0 (19.0–39.0)	24.0 (11.0–41.0)	< 0.001
Follow-up period				0.012
Before COVID-19	211 (36.5%)	71 (29.7%)	140 (41.3%)	
During COVID-19	234 (40.5%)	111 (46.4%)	123 (36.3%)	
Overlap between before and during the COVID-19 pandemic	133 (23.0%)	57 (23.8%)	76 (22.4%)	

*Adherence* is defined by patients who completed baseline and all five ePRO follow-up assessments during the 12-month period following their therapy. BMI = body mass index, DCIS = ductal carcinoma in situ, ePRO = electronic patient-reported outcome, IQR = interquartile range (25th – 75th percentiles), SD = standard deviation

### 12-month adherence and response rate of using ePROs after therapy

**Fig. 2:**
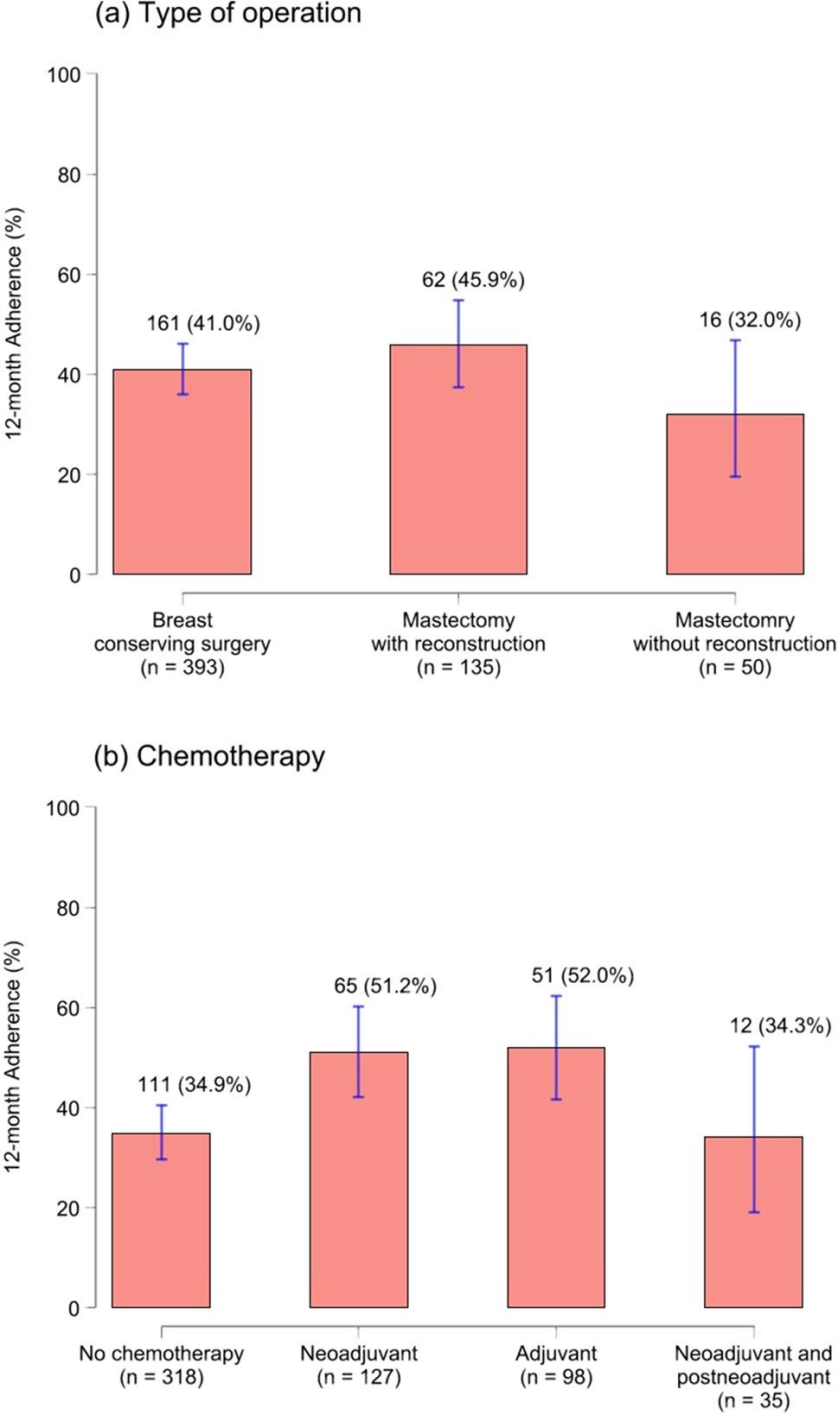
12-month adherence by type of surgery (**a**) and chemotherapy (**b**). Data was presented with percentage (number of cases/total cases). *12-month adherence* is defined by patients who completed baseline and all five ePRO follow-up assessments during the 12-month period following their therapy

The 12-month adherence and response rate of using ePROs post-therapy were investigated among 578 patients. Among the cohort, 239 patients (41.3%, 95%CI: 37.3–45.5%) completed all questionnaires during the 12-month follow-up period. Notably, 45.9% of patients who underwent mastectomy with reconstruction maintained adherence, while 41.0% of those who underwent breast-conserving surgery did so (Fig. 2a). Furthermore, patients who received adjuvant chemotherapy exhibited the highest adherence rate (52.0%, 51/98 cases), followed by those who received neoadjuvant chemotherapy (51.2%) (Fig. 2b).

In terms of response rate trends throughout the follow-up period, almost 70% of patients completed their initial ePRO assessment at 6 weeks, with this rate progressively increasing to 75–80% over the subsequent 12-month period. Notably, except for patients who underwent mastectomy without reconstruction, the average response rates remained consistently above 70% throughout the entire 12-month follow-up period (Supplementary Information Fig. S1).

### Patient characteristics associated with non-adherence

**Fig. 3:**
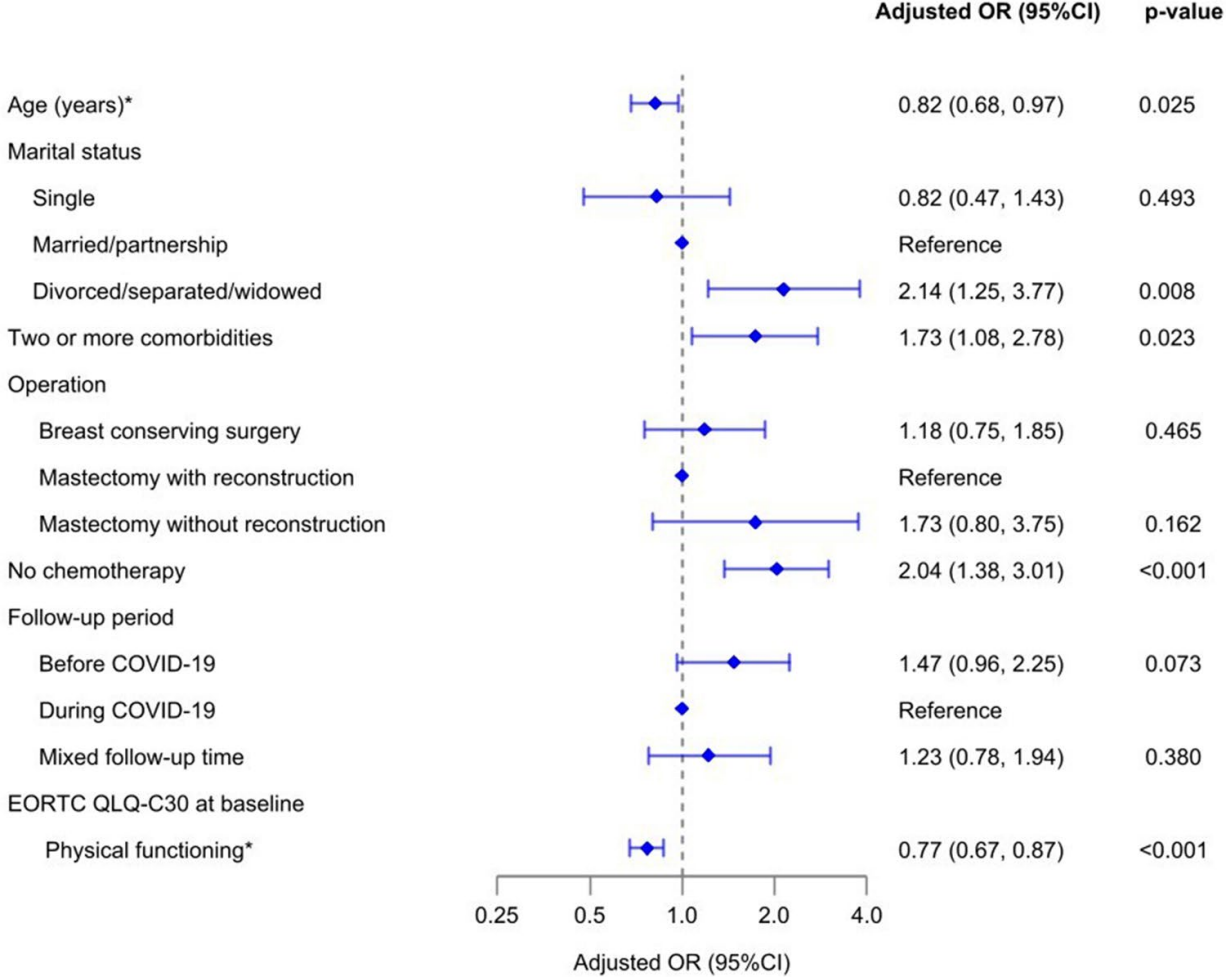
Factors associated with non-adherence to ePRO follow-up in routine care of breast cancer and DCIS patients. * factor is a continuous variable and the value changes by 10 units. Two or more comorbidities compared to less than two comorbidities (reference) and no chemotherapy compared to any chemotherapy

To identify factors linked with non-adherence to ePRO follow-up over a 12-month period, we performed both bivariate (Supplementary Information Table S2) and multivariable analysis. Our final model demonstrated adequate fit (goodness-of-fit test: *p*-value = 0.389). The results showed that with every ten-year increase in age, the odds of non-adherence decreased by 18% (aOR = 0.82, 95% CI: 0.68–0.97). Furthermore, patients who were divorced, separated, or widowed had 2.14 times higher odds of non-adherence (95%CI: 1.25–3.77) compared to those who were married or in a partnership. Patients who underwent breast-conserving surgery or mastectomy without reconstruction exhibited higher likelihoods of non-adherence compared to those who received mastectomy with reconstruction (aOR = 1.18, 95% CI: 0.75–1.85, and aOR = 1.73, 95% CI: 0.80–3.75, respectively). Moreover, patients who did not undergo chemotherapy were more likely to be non-adherent compared to those who received any chemotherapy. Additionally, a ten-point increase in baseline EORTC QLQ C-30 physical functioning score was associated with a 23% decrease in the odds of non-adherence (aOR = 0.77, 95% CI: 0.67–0.87). Notably, patients followed up for 12 months before the COVID-19 pandemic presented higher rates of non-adherence compared to those followed up during the pandemic (aOR = 1.47, 95% CI: 0.96–2.25) (Fig. 3).

Additionally, upon categorizing patients based on reference values derived from the EORTC QLQ C-30 scores at baseline, our findings suggest that patients exhibiting higher scores in physical functioning, role functioning, and social functioning, as well as better symptom scores in fatigue, pain, dyspnoea, and financial difficulties at baseline, were more likely to adhere to the follow-up ePROs (Supplementary Information Table S3).

### Impact of the COVID-19 pandemic on the 12-month adherence to ePRO follow-up

For subgroup analysis, we categorized patients into two groups: those who completed their 12-month ePRO follow-up before 22nd of March 2020 (the first lock-down in Germany) (before COVID-19, *n* = 211 cases), and those who completed ePRO follow-up after this date (during COVID-19, *n* = 234 cases) A total of 133 cases were excluded from the subgroup analysis due to overlapped follow-up periods between the time before and during the COVID-19 pandemic.

**Fig. 4:**
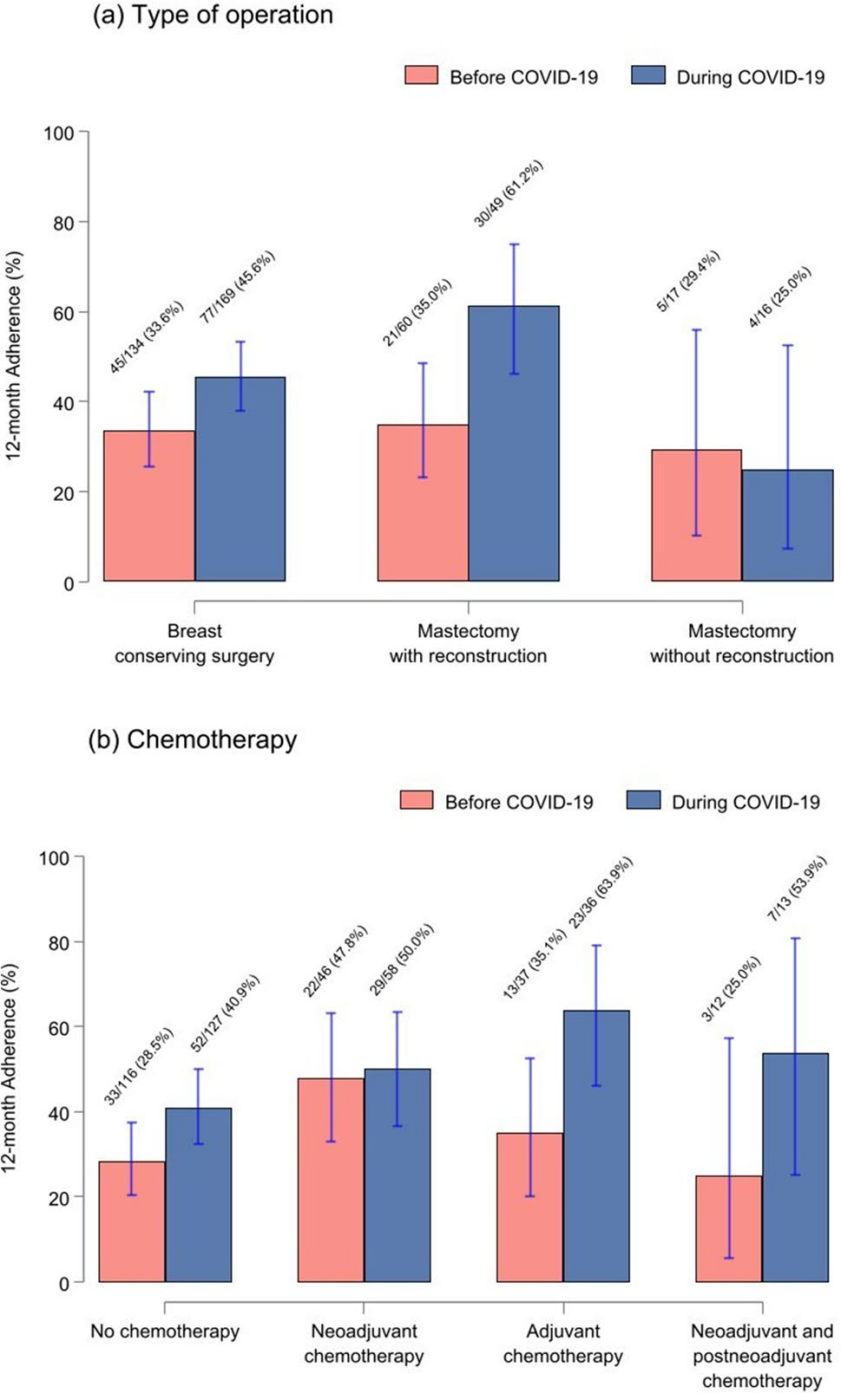
Subgroup analysis: percentage of adherence at baseline and all five follow-up time points by type of surgery (**a**) and chemotherapy (**b**) before and during the COVID-19 pandemic

The 12-month adherence rate was lower before the COVID-19 pandemic compared to during the pandemic (33.6% [71/211] vs. 47.4% [111/234]). Particularly noteworthy is the increase in 12-month adherence among patients who underwent mastectomy with reconstruction during the COVID-19 pandemic, showing a rise of 26.2%, while a slight decline was observed in those who underwent mastectomy without reconstruction (Fig. 4a). Furthermore, patients who received chemotherapy demonstrated higher adherence rates overall during the COVID-19 pandemic compared to before the pandemic, especially in those who received adjuvant chemotherapy (63.9% vs. 35.1%) and those who underwent both neoadjuvant and post-neoadjuvant chemotherapy (53.9% vs. 25.0%) (Fig. 4b).

Furthermore, the response rate at each follow-up time was higher during the COVID-19 pandemic than before the pandemic in the patients who underwent breast-conserving surgery and mastectomy without reconstruction, with the difference ranging from 5 to 10%. However, after the 6-month follow-up, the response rate in patients who underwent mastectomy without reconstruction reversed during the COVID-19 pandemic (Supplementary Information Fig. S2).

## Discussion

Our study reports the results of ePRO follow-up collections in the routine care of invasive early breast cancer and DCIS patients, focusing on adherence in a real-world setting. Adherence to 12-month ePRO follow-up assessments was moderate, with response rates for each follow-up visit ranging between 60 and 80%, comparable to previous publications from real-world settings [16, 18, 23].

It is noteworthy that our study was conducted in a real-world setting, assessing ePROs in clinical routine. Thus, our observed 40% complete adherence rate for the 12-month period (where every questionnaire is answered) is in line with other studies. We also noted a gradual decrease in participation over time in long-term follow-up. Participation rates for ePROs in clinical trial settings are reported to be notably higher (65–75%) [2, 10, 12, 24], although data on complete adherence for long-term follow-up are rarely reported, bringing our average adherence rate fairly close to that of a clinical trial setting.

Factors that may enhance adherence in clinical trial settings include active encouragement from the study team and the use of systems providing two-way communication platforms between caregivers and patients [2, 12, 14]. In addition, some studies allow patients to access their PRO results in real-time and provide recommendations, patient education, or alarm systems [2, 14, 25]. All of these aspects could encourage patients to be more adherent to ePROs. While our electronic questionnaire software currently lacks the capability to provide direct feedback or display PRO scores to patients, we are actively working on methods to provide feedback to participating patients along with specific recommendations based on their ePRO results.

Although the delivery method of questionnaires via email yielded satisfactory response rates for each follow-up visit in our study compared to other studies [26, 27], limitation exist, such as uncertainty regarding email receipt and automated reminder emails potentially being overlooked. Direct patient contact, such as telephone call, may further increase response rates [26] but requires additional resources often unavailable in routine care settings.

Furthermore, our finding that younger patients were more likely to non-adherence compared to older patients contrasts with a previous study associating non-adherence to ePROs with older age [28]. However, this aligns with a previous report from the *PRO routine* project indicating that younger patients exhibit the lowest rate of agreeing to participate in follow-up ePROs [18]. Being younger and healthier might be connected to the assumption of a reduced individual benefit in answering follow-up ePROs, even though it has been reported that younger patients in better health are more willing to participate in electronic surveys than older patients in worse health [29]. Moreover, younger patients undergoing intensive cancer treatments may experience technology fatigue, as evidenced by a recent study where patients with poor adherence perceived ePROs as less reflective of their current health status when the assessments were complex or difficult to understand, and reporting symptoms frequently was perceived as exhausting [28].

While previous studies attributed non-adherence to factors such as low levels of education and preference for paper-based questionnaires [19, 30, 31], we could not confirm these associations. In addition, low adherence has been linked to a limited ability to use digital technology [28, 30], yet our findings indicate the opposite. This discrepancy might be attributed to the email-based nature of our ePRO follow-up, conducted under the condition of consent for participation, and the setting of a tertiary care center at a large university in a metropolitan area.

In line with previous studies [19, 29, 32], we also observe an association between low physical functioning scores of the EORTC QLQ C-30 at baseline and non-adherence in follow-up. Low scores of physical functioning have been reported to correlate with the severity of illness and early decease [32].

Adherence and response rates varied according to the type of surgery, with the lowest rate observed in patients who underwent mastectomy without reconstruction. This phenomenon might be attributable to the relatively small number of patients in this cohort and the likelihood that they have either more advanced disease or poorer overall health compared to those treated with breast-conserving surgery [33]. Patients who underwent mastectomy without reconstruction exhibited a higher tumor stage and lower average physical functioning score at baseline (60% tumor stage 2 or 3) compared to patients who underwent other operations in our sample.

Our findings support to the notion that a higher disease burden or presence of more comorbidities may reduce completion rates of ePRO follow-up assessments. However, the notably high response rates observed in patients who underwent breast-conserving surgery and mastectomy with reconstruction align well with findings from previous studies [34, 35]. Additionally, not receiving chemotherapy emerges as a factors associated with non-adherence, contrary to a previous study which found no differences in adherence based on chemotherapy receipt [34]. This discrepancy may stem from the increased frequency of clinic interactions among chemotherapy patients, leading to reinforced engagement with ePROs by the treatment team.

In our analysis, adherence rates were lower compared to other studies that have evaluated ePROs [16, 36]. Nonetheless, considering our study’s limitations, the adherence rates in the PRO Routine follow-up program remain statistically acceptable[37]. Nevertheless, it is imperative for researchers and healthcare providers to recognize the lower adherence to ePRO in purely observational settings within clinical routines, as it may compromise the completeness of ePRO data due to a high rate of missing data. To encourage both patient participation and enhance patient monitoring, we advocate for the incorporation of features such as real-time feedback, such as PRO-reports, and alarm systems for ePRO systems in routine care.

Medical institutions aiming to implement ePROs in real-world settings for long-term follow-up should augment patient education regarding the benefits of utilizing ePROs, coupled with direct assessment of an individual's ePRO progress. This approach serves to enhance patient motivation and engagement with ePRO assessments. Providing visual feedback on ePRO assessment, along with recommendations for self-management of symptoms, may further bolster patient engagement. To better understand dropout, patients should have the option to provide comments on ePROs at each follow-up, as well as the opportunity to articulate reasons for declining further follow-up. Subsequently, clinicians can target specific factors associated with non-adherence to minimize missing data in subsequent statistical analysis.

Unfortunately, we were unable to assess the extent to which physicians utilized ePRO results during the patient visits, nor do we possess information regarding the potential burden on patients to complete ePROs at each follow-up. Additionally, the reasons for dropping out were not systematically evaluated. As our data were prospectively collected as part of routine care, we conducted a nonrandomized, single-institution study encompassing patients receiving various types of therapy. These limitations have to be taken into consideration.

Since during the COVID-19 pandemic the amount of online surveys conducted increased significantly, a study suggests lower response rates due to survey fatigue [38]. Despite an initial enrollment decrease in our PRO routine program during the COVID-19 pandemic, we observed a notable increase in adherence, similar to the findings of a recent study [17]. Possible explanations for this phenomenon include heightened concerned among patients regarding their health status and increased time spent at home during the pandemic [17]. Moreover, this unexpected discovery stimulates reflection on variables that may have contributed to increased adherence, as well as potential to utilize these findings in current practice. Lessons from the pandemic can be used to guide current attempts for improving adherence through the use of digital health technology, as they indicate the possibility and effectiveness of alternate methods to patient care. We can learn how to use digital health technology more effectively to overcome hurdles to adherence, such as limited access to healthcare facilities, or how the usage of PROs affects both patients and physicians. Furthermore, the study highlighted the importance of patient empowerment and education in increasing adherence to ePROs follow-up, underlining the need for personalized support and communication. Additionally, the need of proactive monitoring and early action in sustaining ePRO adherence is highlighted, emphasizing the need for health care provider feedback when using ePROs to detect problems or worsening outcomes in routine care practices.

## Conclusions

The implementation of an ePRO follow-up assessment at the Charité Breast Center represents a significant step in patient care for breast diseases. However, concerns persist regarding patient adherence rates at long-term follow-up, potentially compromising the completeness of ePRO data. Consequently, healthcare providers should plan to provide enough resources to optimize follow-up rate when employing ePROs in real-world settings for long-term monitoring. Incorporating additional features such as real-time PRO reports accessible to patients and alarm-based monitoring systems might enhance adherence. Moreover, targeted efforts to convey the benefits of participating in an ePRO program, particularly among younger patients and those with a high disease burden, are imperative to enhance adherence rates.

### Supplementary Information

Below is the link to the electronic supplementary material.

Supplementary file1 (DOCX 316 KB)

## Data Availability

The data and the analysis code used in this study are available from the corresponding author upon reasonable request.
