# Supplementary material for: Longitudinal assessment of real-world patient adherence: a 12-month electronic patient-reported outcomes follow-up of women with early breast cancer undergoing treatment
Source: Support Care Cancer. 2024 May 14;32(6):344. doi: 10.1007/s00520-024-08547-7 (PMC11090970; doi:10.1007/s00520-024-08547-7)
Supplement: Supplementary file 1 — Supplementary file1 (DOCX 316 KB) [file 520_2024_8547_MOESM1_ESM.docx]

**ORIGINAL ARTICLE**

**Longitudinal Assessment of Real-world Patient Adherence: A 12-Month Electronic Patient-Reported Outcomes Follow-Up of Women with Early Breast Cancer Undergoing Treatment**

**Running title**

**12-month ePRO adherence in breast cancer patient**

Pimrapat Gebert^a,b^, Anna Maria Hage^c^, Jens-Uwe Blohmer^c^, Robert Roehle^a,b^ and Maria Margarete Karsten^c,^*

^a^ Berlin Institute of Health at Charité –Universitätsmedizin Berlin, Germany

^b^ Charité – Universitätsmedizin Berlin, corporate member of Freie Universität Berlin and Humboldt-Universität zu Berlin, Institute of Biometry and Clinical Epidemiology, Germany

^c^ Charité – Universitätsmedizin Berlin, Corporate Member of Freie Universität Berlin and Humboldt Universität zu Berlin, Department of Gynecology with Breast Center, Germany

***Corresponding author**

PD Dr. med. Maria Margarete Karsten

Charité – Universitätsmedizin Berlin

Department of Gynecology with Breast Center

Charitéplatz 1

10117 Berlin, Germany

Phone: +49 30 450 664 279

Fax: +49 30 450 527 937

Email: [maria-margarete.karsten@charite.de](mailto:maria-margarete.karsten@charite.de)

**Table S1** Follow-up pathways and time points during the first 12 months after start of therapy in the *PRO routine* program

|  | Baseline | Follow-up time points | | | | | |
| --- | --- | --- | --- | --- | --- | --- | --- |
|  | (before therapy) | 2-week | 6-week | 3-month | 6-month | 9-month | 12-month |
| Breast cancer/DCIS | x |  |  |  |  |  |  |
| Breast conserving surgery | x | 🗶 | 🗶 | 🗶 | 🗶 | 🗶 | 🗶 |
| Chemotherapy | x |  | 🗶 | 🗶 | 🗶 | 🗶 | 🗶 |
| Mastectomy with/without reconstruction | x |  | 🗶 | 🗶 | 🗶 | 🗶 | 🗶 |

All patients receive assessments with EORTC QLQ-C30, EORTC QLQ-BR23, PROMIS-29

Additional PROMs for following pathways:

- Breast conserving surgery: Breast-Q Conserving at 2-weeks and 12-months
- Mastectomy with/without reconstruction: Breast-Q Reconstruction at 12-months

**Table S2** Bivariate and multivariable analyses for factors associated with non-adherence to ePROs follow-up

| **Factors** | **Bivariate analysis** | | **Multivariable analysis**  **(final model)** | |
| --- | --- | --- | --- | --- |
|  | **Crude OR (95%CI)** | **p-value** | **Adjusted OR (95%CI)** | **p-value** |
| **Age (years)*** | 1.07 (0.93, 1.22) | 0.368 | 0.82 (0.68, 0.97) | 0.025 |
| **Education level** |  |  |  |  |
| low | 2.87 (1.22, 6.76) | 0.016 |  |  |
| middle | 1.16 (0.78, 1.71) | 0.463 |  |  |
| high | 1.00 |  |  |  |
| **Marital status** |  |  |  |  |
| Single | 0.91 (0.54, 1.53) | 0.729 | 0.82 (0.47, 1.43) | 0.493 |
| Married/partnership | 1.00 |  | 1.00 |  |
| Divorced/separated/widowed | 1.99 (1.18, 3.37) | 0.010 | 2.14 (1.22, 3.77) | 0.008 |
| **Two or more comorbidities** | 2.06 (1.35, 3.15) | 0.001 | 1.73 (1.08, 2.78) | 0.023 |
| **Self-assessment of difficulties completing the baseline PRO questionnaires**  (1=not difficult – 6=very difficult) | 1.18 (1.01, 1.38) | 0.037 |  |  |
| **Preference for paper-based PRO questionnaires at baseline** (yes/no) | 1.36 (0.86, 2.13) | 0.184 |  |  |
| **First breast cancer disease** | 0.76 (0.46, 1.26) | 0.288 |  |  |
| **Tumour stage (AJCC)** |  |  |  |  |
| 0 | 1.45 (0.72, 2.92) | 0.303 |  |  |
| 1 | 1.53 (0.78, 2.98) | 0.214 |  |  |
| 2 | 1.03 (0.52, 2.02) | 0.932 |  |  |
| 3 | 1.00 |  |  |  |
| **Type of surgery** |  |  |  |  |
| Breast-conserving surgery | 1.22 (0.83, 1.81) | 0.315 | 1.18 (0.75, 1.85) | 0.465 |
| Mastectomy with reconstruction | 1.00 |  | 1.00 |  |
| Mastectomy without reconstruction | 1.80 (0.91, 3.58) | 0.091 | 1.73 (0.80, 3.75) | 0.162 |
| **Any chemotherapy (no/yes)** | 1.81 (1.29, 2.53) | 0.001 | 2.04 (1.38, 3.01) | <0.001 |
| **Any target therapy (no/yes)** | 1.12 (0.70, 1.81) | 0.639 |  |  |
| **Any radiology therapy (no/yes)** | 1.11 (0.77, 1.60) | 0.564 |  |  |
| **Any hormon therapy (no/yes)** | 1.18 (0.84, 1.67) | 0.341 |  |  |
| **Follow-up period** |  |  |  |  |
| Before COVID-19 | 1.78 (1.21, 2.61) | 0.003 | 1.47 (0.96, 2.25) | 0.073 |
| During COVID-19 | 1.00 |  | 1.00 |  |
| Overlap between before and during the COVID-19 pandemic | 1.20 (0.78, 1.85) | 0.398 | 1.23 (0.78, 1.94) | 0.380 |
| **EORTC QLQ C-30 at baseline*** |  |  |  |  |
| Global health status/QoL | 0.92 (0.85, 1.00) | 0.047 |  |  |
| **Functioning scales** |  |  |  |  |
| Physical functioning | 0.74 (0.66, 0.84) | <0.001 | 0.77 (0.67, 0.87) | <0.001 |
| Role functioning | 0.91 (0.85, 0.98) | 0.011 |  |  |
| Emotional functioning | 0.98 (0.92, 1.05) | 0.589 |  |  |
| Cognitive functioning | 0.92 (0.85, 0.99) | 0.020 |  |  |
| Social functioning | 0.96 (0.90, 1.01) | 0.141 |  |  |
| **Symptom scales/items** |  |  |  |  |
| Fatigue | 1.11 (1.04, 1.19) | 0.001 |  |  |
| Nausea and vomitting | 1.09 (0.95, 1.25) | 0.205 |  |  |
| Pain | 1.13 (1.06, 1.22) | <0.001 |  |  |
| Dyspnoea | 1.11 (1.04, 1.19) | 0.003 |  |  |
| Insomnia | 1.03 (0.98, 1.08) | 0.208 |  |  |
| Appetite loss | 1.03 (0.97, 1.10) | 0.343 |  |  |
| Constipation | 1.12 (1.02, 1.23) | 0.021 |  |  |
| Diarrhoea | 0.93 (0.87, 1.00) | 0.067 |  |  |
| Financial difficulties | 1.17 (1.08, 1.27) | <0.001 |  |  |

*continuous variables and the unit changes by 10 units.

Increased values of global health status/QoL and functional sub-scales in the EORTC QLQ-C30 indicate a better quality of life, while increased values of symptom sub-scales indicate a poorer quality of life.

**Table S3** Adherence comparison based on EORTC QLQ C-30 scores relative to baseline reference scores

| **EORTC QLQ C-30**  **at baseline** | **Total**  **(n = 578)** | **Adherence**  **(n = 239)** | **Non-adherence**  **(n = 339)** | **Crude RR (95%CI)** | **p-value** |
| --- | --- | --- | --- | --- | --- |
| Global health status/QoL |  |  |  |  |  |
| ≥ 62.70 (+) | 329 | 148 (45.0%) | 181 (55.0%) | 1.19 (0.97, 1.45) | 0.096 |
| < 62.70 (-) | 240 | 91 (37.9%) | 149 (62.1%) | Reference |  |
| Physical functioning |  |  |  |  |  |
| ≥ 84.42 (+) | 393 | 192 (48.9%) | 201 (51.1%) | 1.83 (1.40, 2.38) | <0.001 |
| < 84.42 (-) | 176 | 47 (26.7%) | 129 (73.3%) | Reference |  |
| Role functioning |  |  |  |  |  |
| ≥ 85.85 (+) | 362 | 168 (46.4%) | 194 (53.6%) | 1.35 (1.09, 1.68) | 0.007 |
| < 85.85 (-) | 207 | 71 (34.3%) | 136 (65.7%) | Reference |  |
| Social functioning |  |  |  |  |  |
| ≥ 76.77 (+) | 331 | 151 (45.6%) | 180 (54.4%) | 1.23 (1.01, 1.51) | 0.043 |
| <76.77 (-) | 238 | 88 (37.0%) | 150 (63.0%) | Reference |  |
| Emotional functioning |  |  |  |  |  |
| ≥ 81.78 (+) | 318 | 136 (42.8%) | 182 (57.2%) | 1.04 (0.86, 1.27) | 0.678 |
| < 55.85 (-) | 251 | 103 (41.0%) | 148 (59.0%) | Reference |  |
| Cognitive functioning |  |  |  |  |  |
| ≥ 81.78 (+) | 386 | 172 (44.6%) | 214 (55.4%) | 1.22 (0.98, 1.52) | 0.081 |
| < 81.78 (-) | 183 | 67 (36.6%) | 116 (63.4%) | Reference |  |
| Fatigue |  |  |  |  |  |
| ≤ 29.52 (+) | 296 | 149 (50.3%) | 147 (49.7%) | 1.58 (1.29, 1.94) | <0.001 |
| > 29.52 (-) | 282 | 90 (31.9%) | 192 (68.1%) | Reference |  |
| Nausea and vomitting |  |  |  |  |  |
| ≤ 5.67 (+) | 453 | 194 (42.8%) | 259 (57.2%) | 1.19 (0.92, 1.54) | 0.185 |
| > 5.67 (-) | 125 | 45 (36.0%) | 80 (64.0%) | Reference |  |
| Pain |  |  |  |  |  |
| ≤ 30.7 (+) | 352 | 164 (46.6%) | 188 (53.4%) | 1.40 (1.13, 1.74) | 0.002 |
| > 30.7 (-) | 226 | 75 (33.2%) | 151 (66.8%) | Reference |  |
| Dyspnoea |  |  |  |  |  |
| ≤ 15.82 (+) | 382 | 173 (45.3%) | 209 (54.7%) | 1.34 (1.07, 1.68) | 0.010 |
| > 15.82 (-) | 196 | 66 (33.7%) | 130 (66.3%) | Reference |  |
| Insomnia |  |  |  |  |  |
| ≤ 39.64 (+) | 368 | 157 (42.7%) | 211 (57.3%) | 1.09 (0.89, 1.34) | 0.400 |
| > 39.64 (-) | 210 | 82 (39.0%) | 128 (61.0%) | Reference |  |
| Appetite loss |  |  |  |  |  |
| ≤ 16.19 (+) | 393 | 164 (41.7%) | 229 (58.3%) | 1.03 (0.83, 1.27) | 0.787 |
| > 16.19 (-) | 185 | 75 (40.5%) | 110 (59.5%) | Reference |  |
| Constipation |  |  |  |  |  |
| ≤ 6.54 (+) | 471 | 207 (43.9%) | 264 (56.1%) | 1.47 (1.08, 2.00) | 0.014 |
| > 6.54 (-) | 107 | 32 (29.9%) | 75 (70.1%) | Reference |  |
| Diarrhoea |  |  |  |  |  |
| ≤ 9.05 (+) | 454 | 182 (40.1%) | 272 (59.9%) | 0.87 (0.70, 1.09) | 0.226 |
| > 9.05 (-) | 124 | 57 (46.0%) | 67 (54.0%) | Reference |  |
| Financial difficulties |  |  |  |  |  |
| ≤ 10.47 (+) | 437 | 200 (45.8%) | 237 (54.2%) | 1.65 (1.24, 2.20) | 0.001 |
| > 10.47 (-) | 141 | 39 (27.7%) | 102 (72.3%) | Reference |  |

The relative risk (RR) is estimated using a generalized linear model with a binomial family and a log link function, the outcome is adherence. A plus sign (+) indicates that the scores are better than the average reference scores at baseline, while a minus sign (-) indicates that the scores are worse than the average reference scores at baseline.

**
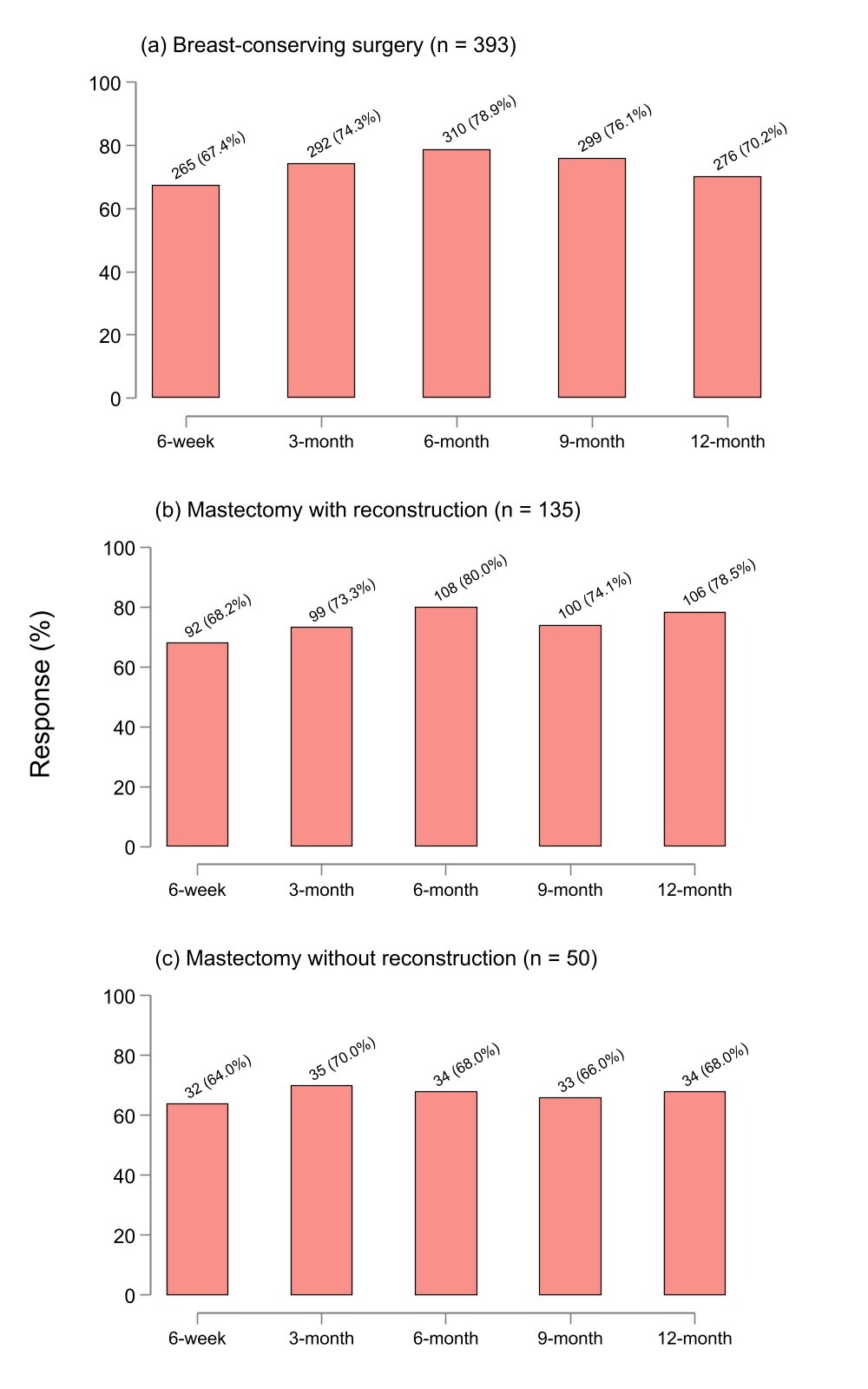
**

**Fig. S1** Percentage of response at each follow-up time point by type of surgery


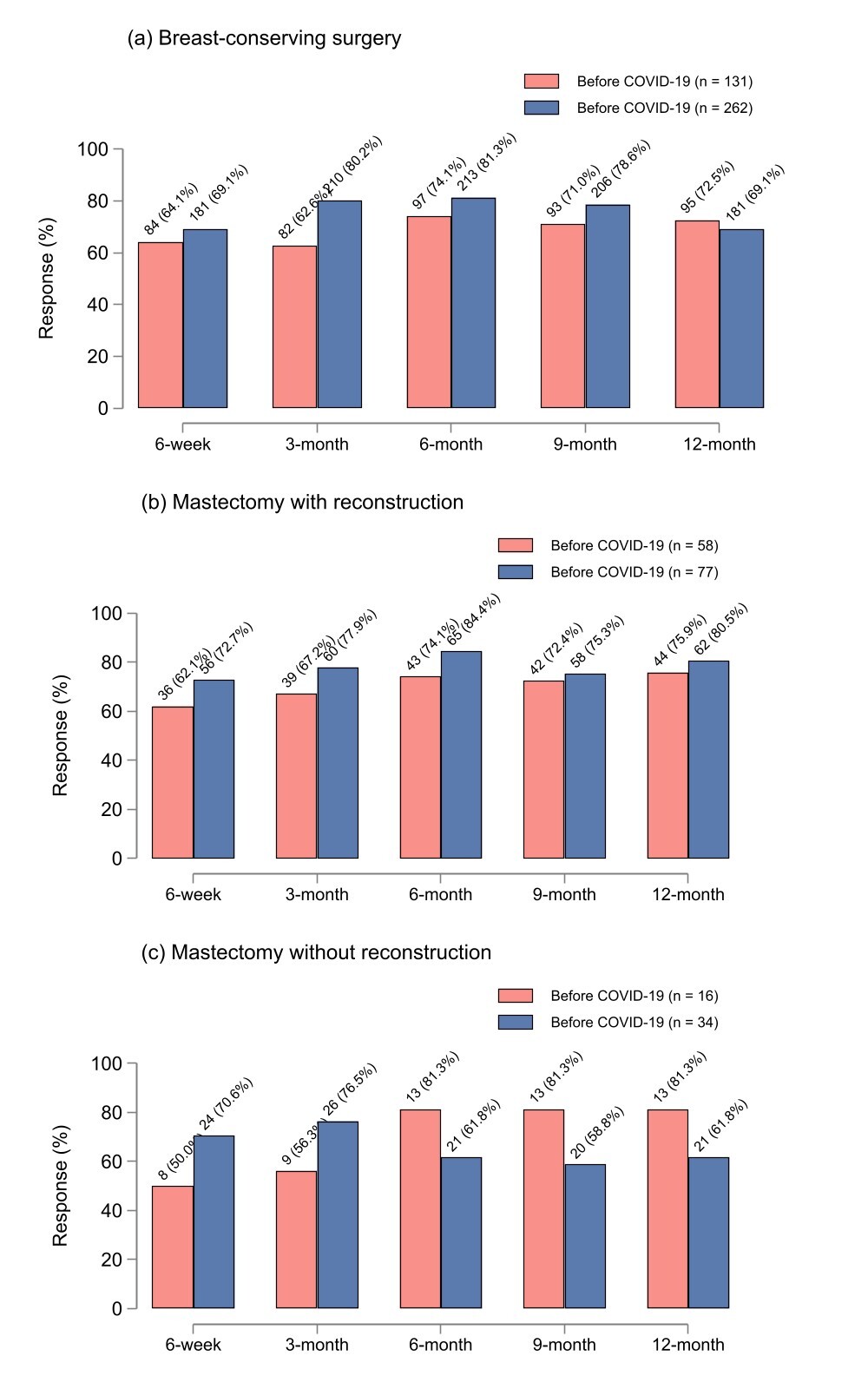


**Fig. S2** Subgroup analysis: percentage of response at each follow-up time point by type of surgery before and during the COVID-19 pandemic
